# Highly Thermotolerant SARS-CoV-2 Vaccine Elicits Neutralising Antibodies against Delta and Omicron in Mice

**DOI:** 10.3390/v14040800

**Published:** 2022-04-13

**Authors:** Petrus Jansen van Vuren, Alexander J. McAuley, Michael J. Kuiper, Nagendrakumar Balasubramanian Singanallur, Matthew P. Bruce, Shane Riddell, Sarah Goldie, Shruthi Mangalaganesh, Simran Chahal, Trevor W. Drew, Kim R. Blasdell, Mary Tachedjian, Leon Caly, Julian D. Druce, Shahbaz Ahmed, Mohammad Suhail Khan, Sameer Kumar Malladi, Randhir Singh, Suman Pandey, Raghavan Varadarajan, Seshadri S. Vasan

**Affiliations:** 1 Australian Centre for Disease Preparedness, Commonwealth Scientific and Industrial Research Organisation, Geelong, VIC 3220, Australia; petrus.jansenvanvuren@csiro.au (P.J.v.V.); alex.mcauley@csiro.au (A.J.M.); nagendra.singanallur@csiro.au (N.B.S.); matthew.bruce@csiro.au (M.P.B.); shane.riddell@csiro.au (S.R.); sarah.goldie@csiro.au (S.G.); sman0040@student.monash.edu (S.M.); simran.chahal@csiro.au (S.C.); trevor.drew@csiro.au (T.W.D.); kim.blasdell@csiro.au (K.R.B.); mary.tachedjian@csiro.au (M.T.); 2 Data61, Commonwealth Scientific and Industrial Research Organisation, Docklands, VIC 3008, Australia; michael.kuiper@data61.csiro.au; 3 Monash Biomedicine Discovery Institute, Monash University, Clayton, VIC 3800, Australia; 4 Victorian Infectious Diseases Reference Laboratory, The Royal Melbourne Hospital and The Peter Doherty Institute for Infection and Immunity, Melbourne, VIC 3000, Australia; leon.caly@mh.org.au (L.C.); julian.druce@mh.org.au (J.D.D.); 5 Molecular Biophysics Unit, Indian Institute of Science, Bengaluru 560012, India; shahbazs@iisc.ac.in (S.A.); mohammadkhan@iisc.ac.in (M.S.K.); sameerm@iisc.ac.in (S.K.M.); varadar@iisc.ac.in (R.V.); 6 Mynvax Private Limited, ES-12, Incubation Centre, Society for Innovation and Development, Indian Institute of Science, Bengaluru 560012, India; randhir.singh@mynvax.com (R.S.); suman.pandey@mynvax.com (S.P.); 7 Department of Health Sciences, University of York, York YO10 5DD, UK

**Keywords:** AlphaFold, biomolecular modelling, COVID-19, Omicron, receptor-binding domain (RBD), SARS-CoV-2, thermotolerant vaccine, vaccine equity, vaccine matching, variants of concern (VOC)

## Abstract

As existing vaccines fail to completely prevent COVID-19 infections or community transmission, there is an unmet need for vaccines that can better combat SARS-CoV-2 variants of concern (VOC). We previously developed highly thermo-tolerant monomeric and trimeric receptor-binding domain derivatives that can withstand 100 °C for 90 min and 37 °C for four weeks and help eliminate cold-chain requirements. We show that mice immunised with these vaccine formulations elicit high titres of antibodies that neutralise SARS-CoV-2 variants VIC31 (with Spike: D614G mutation), Delta and Omicron (BA.1.1) VOC. Compared to VIC31, there was an average 14.4-fold reduction in neutralisation against BA.1.1 for the three monomeric antigen-adjuvant combinations and a 16.5-fold reduction for the three trimeric antigen-adjuvant combinations; the corresponding values against Delta were 2.5 and 3.0. Our findings suggest that monomeric formulations are suitable for upcoming Phase I human clinical trials and that there is potential for increasing the efficacy with vaccine matching to improve the responses against emerging variants. These findings are consistent with *in silico* modelling and AlphaFold predictions, which show that, while oligomeric presentation can be generally beneficial, it can make important epitopes inaccessible and also carries the risk of eliciting unwanted antibodies against the oligomerisation domain.

## 1. Introduction

The novel coronavirus disease 19 (COVID-19) was declared by the World Health Organization (WHO) as a ‘Public Health Emergency of International Concern’ on 30 January 2020 and as a pandemic on 11 March 2020. The WHO originally predicted that COVID-19 would take 4–5 years to control [1], and as we enter the third year of the ongoing pandemic, this target seems tantalisingly close. With already 428 million cases and 5.91 million deaths as of 24 February 2022 [2], vaccination remains the key defence against the severe acute respiratory syndrome coronavirus 2 (SARS-CoV-2).

In the context of vaccination, the WHO has listed four factors—poor coverage (particularly in vulnerable populations), inequitable access, sub-optimal duration of protection post-vaccination (against infection, severe disease and death) and the emergence of variants of concern (VOC)—as the key factors driving the impact of SARS-CoV-2 [3]. The first two factors, poor coverage and inequitable access, are interlinked and impacted by cold-chain storage requirements, while the next two factors, sub-optimal protection and VOC, are also interlinked.

**Figure 1:**
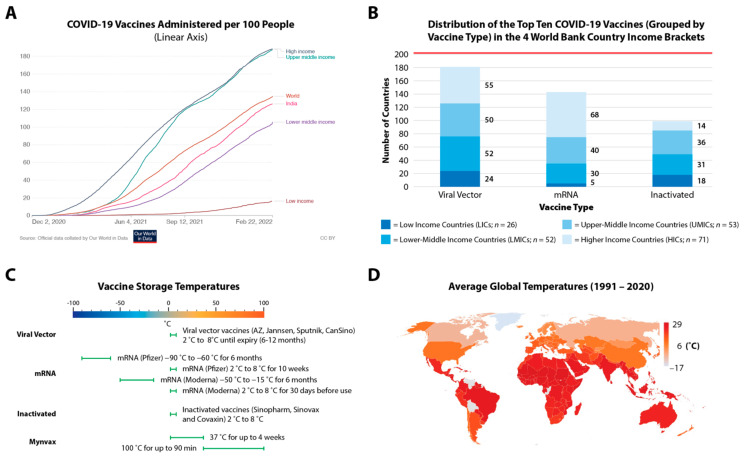
COVID-19 vaccine storage temperature and cold-chain transport affects equitable access. The total doses (including boosters) administered per 100 people is shown in (**A**), while (**B**) shows the top 10 vaccines (grouped into three categories) inequitably distributed across the four World Bank country income brackets. (**C**) Storage temperatures for these top 10 vaccines (grouped under three headings) is compared to Mynvax formulation. (**D**) The average storage temperatures show a high level of correlation with vaccine inequity experienced by LICs and LMICs. In (**B**), the red line denotes the total number of countries used in our analysis (*n* = 202) based on availability of information from Our World in Data. The data in (**A**) was accurate as of 22 February 2022 and in (**B**) was accurate as of 6 February 2022.

Globally, 10.6 billion doses of the COVID-19 vaccines have been administered to date, indicating that 4.4 billion of the world’s population has been fully vaccinated [2]. However, the reality is that half the world’s population, predominantly in low-income countries (LICs) and lower middle-income countries (LMICs), is still receiving its first dose of COVID-19 vaccines. In contrast, the administration of third doses is well underway in most upper middle-income countries (UMICs) and high-income countries (HICs), with Israel having already giving the fourth dose for its nationals [4]. LICs have administered 13-fold fewer vaccines compared to both UMICs and HICs, while LMICs (including India, which is responsible for 62% of the global vaccine production) are below the world-average (Figure 1A) [5].

Grouping the world’s top-10 most-administered vaccines based on their type—(a) Viral Vector vaccines (AstraZeneca/AZ (Vaxzevria/AZD1222), Janssen, SputnikV/Sputnik Light (Gam-COVID-Vac) and Convidecia/CanSino (AD5-nCOV)), (b) mRNA vaccines (Pfizer (Comirnaty/BNT162b2) and Moderna (Spikevax/mRNA-1273)) and (c) Inactivated vaccines (Sinopharm (BBIBP-CorV/NVSI-06-07), Covaxin (BBV152) and Sinovac (CoronaVac))—we see that the viral vectored and inactivated vaccines (which only require storage at 2–8 °C) are more evenly distributed across the different country income brackets (Figure 1B,C). In contrast, Pfizer requires ultra-cold-chain freezer capacity at −70 °C, and Moderna requires traditional freezer capacity at −20 °C (Figure 1C); these have largely benefited HICs and UMICs, which also have greater manufacturing expertise for mRNA vaccines, highlighting that vaccine inequity and cold storage requirements are highly correlated.

As no country is protected from this virus until all countries are, it is of paramount importance to address this inequity, keep up with the emergence of new variants and improve vaccination rates in LICs and LMICs. These countries are ill-equipped for the financial and logistical burdens of cold-chain transport and storage requirements, which are necessary for all approved COVID-19 vaccines to varying degrees (Figure 1C). This is one of the key contributing factors disproportionately impacting LICs and LMICs where the average temperatures are higher (Figure 1D) [6,7].

At 14.2 doses per 100 people, most people in LICs have not received their first dose; we have ample evidence that a single dose does not adequately prevent infections both at an individual level and regarding community transmission. For instance, with the Pfizer vaccine, protection against symptomatic COVID-19 was only at 52% twelve days after the first dose [8]. This problem has been exacerbated by the emergence of variants—five of which have been declared to be of concern, and newer ones could arise in poorly vaccinated regions. 

The duration of protection is even lower with the recent Delta and Omicron; our recent study with AstraZeneca, Moderna and Pfizer showed that at least a third dose is required to generate sufficient neutralising antibody titres against these two VOC [9]. Therefore, we urgently need next-generation vaccines (as described in this paper), which are not only highly effective against all existing and emerging variants of SARS-CoV-2 but are also thermostable and do not have cold-chain transport and storage requirements.

## 2. Materials and Methods

**Figure 2:**
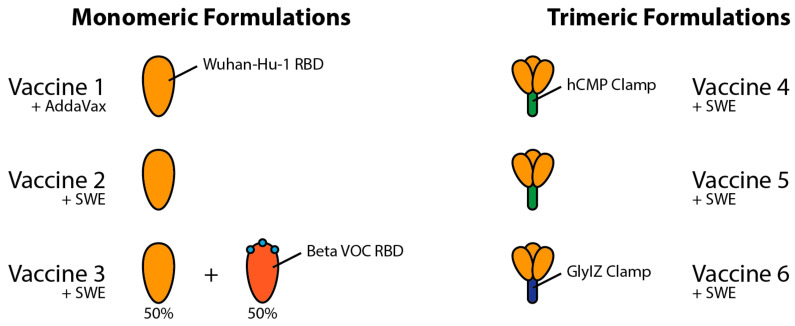
Schematic diagram of vaccine formulations 1–6 evaluated by this study (c.f. Table 1). Supplementary Text A  contains further amino acid information.

In this paper, we analyse the serological responses of the VOC in mice for six vaccine formulations developed by Mynvax Private Limited, one of which is to be selected for upcoming Phase I human clinical trials. The underlying antigens are highly thermo-tolerant monomers (Vaccines 1–3, c.f. Section 2.1) or trimers (Vaccines 4–6, c.f. Section 2.2) as shown in Figure 2. Mynvax has previously shown that its receptor-binding domain derivatives can withstand temperatures up to 100 °C for 90 min and 37 °C for four weeks [10,11,12]; therefore, this paper will not go into proving thermostability. Details of mice immunisation are presented in Section 2.3, while Section 2.4 and Section 2.5 describe the materials and methods for live virus neutralisation assays. Section 2.6 and Section 2.7, respectively, describe the in silico and statistical methods used to interpret the results.

### 2.1. Thermostable Monomer-Adjuvant Formulations

The RBD of SARS-CoV-2 and its mutants were cloned in the pcDNA3.4 vector for expression in Expi293F cells as described previously [10,11]. After transfection and protein purification, the mRBD1-3.2 RBD protein was obtained at a yield of ~180 mg/L. These mammalian cell-expressed wild-type (WT) and mRBD1-3.2 RBD at 0.2 mg/mL of PBS or adjuvants (SWE) were stored at 4 °C and 45 °C for up to 28 days. Aliquots were taken at regular intervals and diluted in PBS to a final concentration of 100 nM, and the amount of folded protein remaining was estimated using surface plasmon resonance (SPR). 

In another set of experiments, following dialysis against water and lyophilisation, RBD was subjected to thermal incubation at 37 °C for up to 30 days in individual aliquots. At each time point, aliquots were returned to 4 °C. Prior to SPR and differential scanning fluorimetry (DSF), samples were resolubilised in PBS at concentrations of 100 nM and 0.2 mg/mL, respectively. SPR binding to immobilised angiotensin-converting enzyme 2 (ACE-2) hFc and DSF were performed as described previously.

The following ‘Mynvax’ protein sub-unit monomer vaccine formulations 1–3 were prepared, as follows:

Vaccine 1: mRBD1-3.2 is a stabilised multi-mutant of RBD (residues 331–532) of SARS-CoV-2 Spike (S) protein. It contains A348P, Y365W and P527L stabilising mutations [10,11]. The protein was expressed in mammalian Expi293F cells (Thermo Fisher Scientific, Cat no. A14527) and purified using Ni-NTA (GE Healthcare, Uppsala, Sweden, Cat no. 17531802) affinity purification. This antigen has been shown to be stable at 37 °C for up to a month without any reduction in the amount of folded fraction. The protein antigen was adjuvanted with AddaVax (1:1 *v*/*v*; vac-adx-10, InvivoGen, San Diego, Caifornia, USA) for immunisation.

Vaccine 2: The stabilised antigen mRBD1-3.2 was adjuvanted with a squalene-in-water emulsion adjuvant (SWE)—a GMP-grade adjuvant equivalent to MF59 (Sepivac SWE, 1:1 *v*/*v*; Cat. No. 80748, SEPPIC SA, France)—for immunisation.

Vaccine 3: A formulation of equal amounts (20 µg each) of mRBD1-3.2 and mRBD1-3.2-beta was adjuvanted with SWE (1:1 *v*/*v*) for immunisation. mRBD1-3.2-beta was generated in the background of stabilised mRBD1-3.2 (A348P, Y365W and P527L), and it has three important mutations (K417N, E484K and N501Y) present in the RBD of the Beta (B.1.351) VOC.

### 2.2. Trimeric Human Cartilage Matrix Protein (hCMP) Formulations

Malladi et al. [10] previously designed a monomeric glycan engineered derivative of the receptor-binding domain termed mRBD (residues 332–532 possessing an additional glycosylation site at N532) that induced neutralising antibodies in guinea pig immunisations. Oligomerisation of native antigens was expected to induce higher titres of binding and neutralising antibodies; therefore, the mRBD was fused to the disulphide linked trimerisation domain derived from hCMP (residues 298–340). We hypothesised that RBD fused to the hCMP trimerisation domain (residues 298–340) would elicit higher neutralising antibody titres relative to the corresponding monomer. The ‘Mynvax’ protein sub-unit trimer vaccine formulations 4–6 used in this work were as follows:

Vaccine 4: The WT RBD (residues 332–532) was fused to the C-terminus of the disulphide linked trimerisation domain derived from hCMP, and the resultant construct was called hCMP-mRBD [12]. The protein was expressed in mammalian Expi293F cells (Thermo Fisher Scientific, Carlsbad, CA, USA, Cat no. A14527) and purified using Ni-NTA (GE Healthcare, Uppsala, Sweden, Cat no. 17531802) affinity purification. This antigen was adjuvanted with SWE (1:1 *v*/*v*) for immunisation. This antigen was shown to be highly thermotolerant and remained folded for up to a month when lyophilised powder was incubated at 37 °C. The protein was also tolerant to transient thermal stress to 100 °C for up to 90 min.

Vaccine 5: The above-mentioned hCMP-mRBD antigen when expressed from Stable CHO (Thermo Fisher Scientific, Carlsbad, CA, USA, Cat no. R75807) cell lines was named as hCMP-mRBD-CHO. This antigen was adjuvanted with SWE (1:1 *v*/*v*) for immunisation.

Vaccine 6: The glycosylated synthetic trimerisation domain IZN4 [13] was fused at the C-terminus of WT RBD (residues 332–532) [12]. The presence of glycosylation in the trimerisation domain reduced the immune response against the scaffold. This antigen was adjuvanted with SWE (1:1 *v*/*v*) for immunisation.

### 2.3. Mouse Immunisation

The purified protein antigens (20 µg per animal) were formulated with the ‘MF59′ equivalent squalene-based oil-in-water emulsion AddaVax or SWE adjuvants. The formulations were administered to 6–8-week-old female BALB/c mice (*n* = 5) via intramuscular injection, on Day 0 (prime), Day 21 (first boost) and Day 42 (second boost). The protein antigen (20 µg in 50 µL) was diluted with the adjuvant (50 µL) before immunisation. Pre-bleed serum was collected two days before immunisation, and at two weeks post prime and boosts, serum was collected to estimate the titres of elicited IgG antibodies. The study was conducted at the Central Animal Facility, Indian Institute of Science, Bangalore, India. All animal studies were approved by the Institutional Animal Ethics Committee (IAEC no. CAF/ETHICS/799/2020). The mouse samples were imported into ACDP, Geelong, Australia and were gamma irradiated on entry per import permit conditions.

### 2.4. SARS-CoV-2 Isolation and Stocks

Three SARS-CoV-2 isolates viz., VIC31-D614G (hCoV-19/Australia/VIC31/2020, containing the D614G mutation) and the two variants of concern (VOC) Delta (hCoV-19/Australia/VIC18440/2021) and Omicron BA.1.1 (hCoV-19/Australia/VIC28585/2021), were kindly provided by Drs Caly and Druce at the Victorian Infectious Diseases Reference Laboratory (VIDRL; Melbourne, Australia). Virus stocks were propagated and titrated in Vero E6 cells (American Type Culture Collection (ATCC), Manassas, VA, USA) prior to use as described in Malladi et al. [12], with TCID_50_ titres calculated using the method of Spearman and Kärber [14].

The identity of virus stocks was confirmed by next-generation sequencing using a MiniSeq platform (Illumina, Inc.; San Diego, CA, USA). RNA was purified from Trizol-inactivated material using a Direct-zol RNA Miniprep kit (Zymo Research; Irvine, CA, USA). Purified RNA was further concentrated using an RNA Clean-and-Concentrator kit (Zymo Research). RNA was converted to double-stranded cDNA, ligated and then isothermally amplified using a QIAseq FX single cell RNA library kit (Qiagen, Hilden, Germany). Fragmentation and dual-index library preparation was conducted with an Illumina DNA Prep, Tagmentation Library Preparation kit. 

The average library size was determined using a Bioanalyser (Agilent Technologies; San Diego, CA, USA) and quantified with a Qubit 3.0 Fluorometer (Invitrogen; Carlsbad, CA, USA). Denatured libraries were sequenced on an Illumina MiniSeq using a 300-cycle Mid-Output Reagent kit as per the manufacturer’s protocol. Paired-end Fastq reads were trimmed for quality and mapped to the published sequence for the SARS-CoV-2 reference isolate Wuhan-Hu-1 (RefSeq: NC_045512.2) using CLC Genomics Workbench version 21 from which consensus sequences were generated. Stocks were confirmed to be free from contamination by adventitious agents by analysis of reads that did not map to SARS-CoV-2 or cell-derived sequences.

### 2.5. Live Virus Neutralisation Assays

Virus neutralisation assays (VNT) were carried out using VeroE6 cells as described previously [12]. Briefly, each serum sample was diluted 1:80 (or 1:160 where sample volume was insufficient for 1:80) in DMEM-D in a deep-well plate, followed by a two-fold serial dilution up to 1:10,240 (or 1:20,480 where 1:160 was the starting dilution). The dilution series for each serum sample were dispensed into rows of a 96-well plate for a total volume of 50 µL per well and triplicate wells per sample dilution. For the serum-containing wells, 50 µL virus diluted in medium to contain approximately 100 TCID_50_ (checked by back-titration) was added to each well. A positive control serum was prepared from pooled ferret sera generated in a previous study [15] and was successfully used in subsequent studies, e.g., [9,11,12] and in this one to confirm assay reproducibility.

The plates were incubated at 37 °C/5% CO_2_ for 1 h to allow neutralisation complexes to form between the antibodies present in the sera and the virus. At the end of the incubation, 100 µL VeroE6 cells (2 × 10^4^ cells/well) were added to each well and the plates were returned to the incubator for 4 days. Each well was scored for the presence of viral CPE, readily discernible on Day 4 post-infection, with SN_50_ neutralisation titres calculated using the Spearman–Kärber formula [14] and transformed to log_2_ values for analysis. 

Replicates that did not show neutralisation at the lowest dilution tested (i.e., 1:80 or 1:160) were scored as ≤1:57 and ≤1:113, respectively. For statistical analysis, all samples yielding results below the detection limit were assigned a value of 1:57 based on the assumption that samples with a 1:80 starting dilution would have 100% neutralisation at one lower dilution (1:40), thereby, yielding a titre of 1:57 based on the Kärber calculation (please refer to Supplementary Text B).

### 2.6. Modelling of SARS-CoV-2 Spike Protein Based on In Silico Methods

Models of the S protein receptor-binding domains (RBD, residues 332 to 532) of the major variants of concern, (Alpha, Beta, Gamma, Delta and Omicron) as well as the trimeric hCMP construct were built using AlphaFold (version 2.1.1) [16]. Models were inspected visually using VMD [17] to highlight and map the relative positions of mutations. AlphaFold provided consistent models for the RBD domains with high confidence scores; however, it provided multiple conformations for the trimeric constructs.

### 2.7. Statistical Analysis

VNT titre data were analysed and expressed as Log base 2 (Log_2_). The vaccine group means and standard deviations were calculated and expressed as the mean and standard deviation of the mean (SD). Both one-way and two-way ANOVA were used to test the statistical differences between vaccines, antigens and adjuvants. If the ANOVA returned a *p*-value of <0.05, a post hoc with Tukey’s range test (Tukey’s HSD) was performed to measure the pairwise difference and interactions between two variables. All statistical procedures were performed in R [18]) using the ‘*car*’ and ‘*lme*’ libraries.

## 3. Results

The Mynvax vaccine formulations elicited high antibody titres in mice that received prime-boost immunisations on Days 0, 21 and 42 against the wildtype SARS-CoV-2 virus. We previously demonstrated that the ELISA and neutralisation titres are virtually identical post the first and second boosts [11]. Neutralisation assays were performed using VIC31-D614G, Delta and Omicron SARS-CoV-2 variants. 

**Figure 3:**
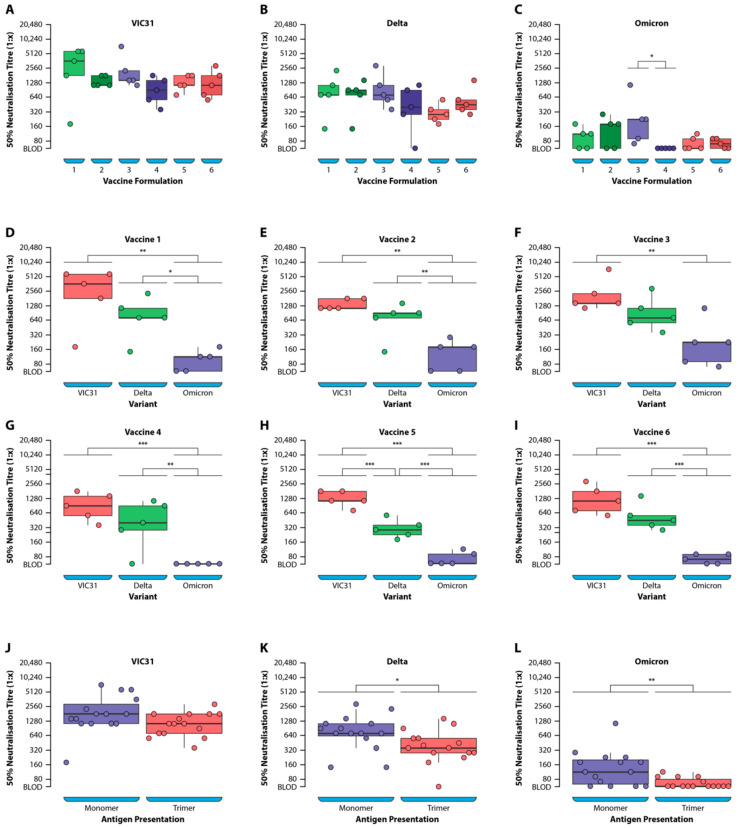
ANOVA analysis of neutralising antibody titres against SARS-CoV-2 VIC31-D614G, Delta and Omicron BA.1.1 variants following mouse immunisation with different vaccine formulations (**A**–**I**) and comparing monomeric to trimeric formulations (**J**–**L**). * *p* < 0.05; ** *p* < 0.01; *** *p* < 0.001.

The raw data for neutralising antibody titres for individual mice as well as the mean values for the positive control serum used in the neutralisation assays are provided in Table 1, including the assignment of individual mice to antigen-adjuvant vaccine formulations. The box plot in Figure 3A–C represent the antibody titres for mice in each group with the median values shown as horizonal lines in the plot. 

As with all neutralising antibody assays, a positive control serum was included in the study as assay control and back titrations were performed on the virus pools used in the assay. All control samples passed the assay criterion and virus back titrations were within acceptable limits.

The mean neutralising antibody titres (Log_2_-transformed) with standard deviations from the means, for the three variants and different vaccine formulations, are presented in Table 2. There was an average 2.7-fold reduction in neutralising titres to Delta compared with VIC31-D614G, whereas an average 15.4-fold reduction was noted for Omicron across all six vaccine formulations. For monomeric formulations the fold reduction values were 2.5 and 14.4 and for trimeric 3.0 and 16.5, to Delta and Omicron, respectively, compared to VIC31-D614G. The fold-reduction for Omicron, compared to Delta, was 5.7- and 6.0-fold for monomeric and trimeric formulations, respectively.

The neutralising antibody titres for the three SARS-CoV-2 variants were compared for the six vaccine groups (Figure 3A–I). There was a reduction in neutralising titres to Delta ranging from 2.1- to 4.2-fold for the six vaccine formulations, when compared to VIC31-D614G, although this reduction was only statistically significant for Vaccine 5 (4.2-fold, *p* < 0.01) (Table 2 and Table 3). The reduction in neutralisation of Omicron was more pronounced, ranging from a 10.1- to 22.0-fold decrease for the six vaccine formulations, when compared to VIC31-D614G and was statistically significant (*p* < 0.01) for all. Vaccine 3 yielded neutralising titres 10-fold lower than Omicron when compared with VIC31, whereas the other five formulations yielded an average fold decrease of 16.5 (range 11- to 22-fold). The fold-reduction (range 4.2 to 7.6) for Omicron compared to Delta was also statistically significant (*p* < 0.05) for all vaccine formulations except Vaccine 3 (*p* < 0.1).

A summary of the One-way ANOVA analysis (Table 3) shows that only the “Variant” and “Presentation” variables were responsible for statistically significant differences in the neutralising titres noted between different vaccine groups. No significant difference (*p* > 0.05) was noted in the neutralising antibody titres for the three variants following immunisation with the different vaccine formulations, except for Omicron Vaccine 3 vs. Vaccine 4 (*p* < 0.05) (Figure 3A–C; Table 3). 

The mean antibody titres to VIC31-D614G following immunisation with the vaccine formulations comprising antigens presented as monomers (Vaccines 1–3) showed a statistically non-significant (*p* < 0.1) increase when compared to the trimers (Vaccines 4–6) (Figure 3J–L; Table 3). However, a statistically significant increase in the average VNT titres was noted for both Delta (*p* < 0.05) and Omicron (*p* < 0.01) variants when comparing monomeric to trimeric antigen presentation (Figure 3J–L; Table 3). No significant difference (*p* > 0.05) was noted in the neutralising antibodies for all three variants when comparing the six antigens or adjuvants used in vaccine formulations (Table 3).

**Figure 4:**
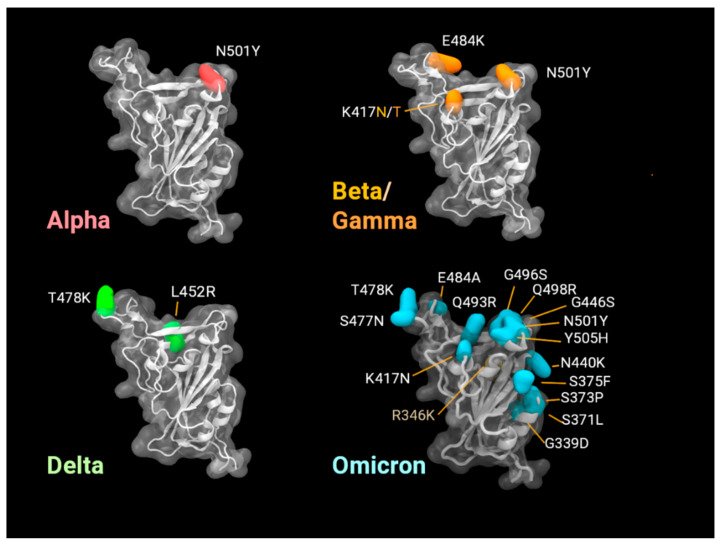
Visualisation of variant mutations in the RBD of the SARS-CoV-2 spike protein (residues 330 to 530). Omicron BA.1.1 is notable for the numbers of new mutations in this region (15 compared to 3 in Beta/Gamma and 1 in Alpha). Our isolate also included the mutation R346K. Beta and Gamma variants present similar RBDs; differing at position 417, by Asn (N).

**Figure 5:**
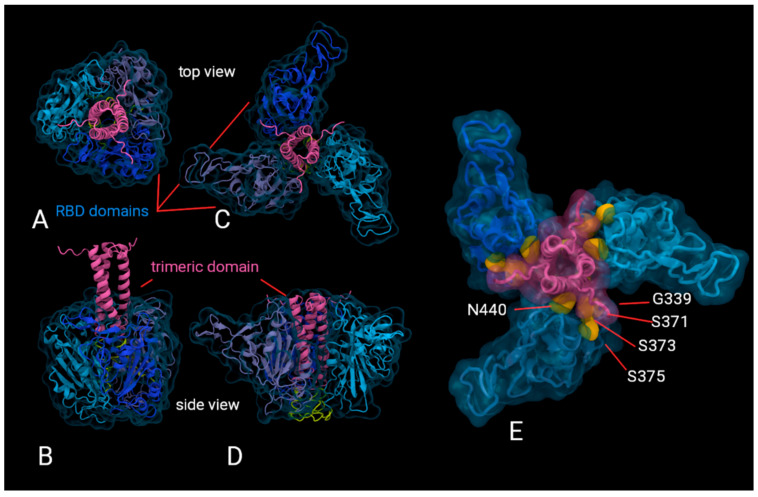
Visualisations of the AlphaFold predictions of the trimeric construct. (**A**,**B**) show the top and side views of one probable structure; (**C**,**D**) of another. RBD domains are shown in blues, while the trimerisation domain is shown in pink. (**E**) shows (**C**) in greater detail, highlighting in yellow the buried G339, S371, S373, S375 and N440 residues, which are mutation sites in Omicron.

In order to understand these experimental results, it was informative to look at predictions of our in silico molecular dynamics (Figure 4) and AlphaFold (Figure 5) modelling. As all six vaccine antigens described in this paper are RBD derivatives, the homology model in Figure 4 shows the mutations in this domain for the five VOC to date; the difference in epitopes for each VOC is highlighted in corresponding colours. Vaccine 3 is matched to Beta; however, this VOC only shares N501Y and K417N mutations with Omicron. In this study, we used Omicron BA.1.1, which has an additional R346K mutation along with a constellation of mutations in S (compared to past VOC, c.f. Figure 4).

AlphaFold predictions of likely structures for the trimeric formulations reveal a high probability of epitopes being buried and inaccessible for recognition by B-cells for antibody development. Figure 5 shows two examples of AlphaFold2 predictions of the trimeric structure as this artificial intelligence software is currently only able to give possible structures for the multimer. That we do not have a definitive structure prediction is not a handicap for our purpose because we are able to show that key neutralising epitopes are inaccessible in each of these probable trimeric structures (showing the G339, S371, S373, S375 and N440 sites mutated in Omicron VOC as an example). We also have a negative stain transmission electron micrograph published elsewhere ([12] (c.f. Figure 1h in that reference)), which shows that the lobed structure shown in Figure 5C–E is more consistent with the experimental data.

## 4. Discussion

The emergence of SARS-CoV-2 VOC with considerable mutations in the spike protein, such as Omicron (BA.1.1), and waning immunity in vaccinated individuals [9,19], highlight the need for improved vaccines that are more easily deployable with fewer logistical constraints. The ability of the Omicron variant to escape neutralisation by a significant proportion of monoclonal antibody cocktails [20,21] as well as serum or plasma from vaccinated and/or infected patients [22,23] further highlights the need for a regular review of the ability of vaccine induced immunity to neutralise circulating variants and potential subsequent vaccine matching. 

The development of highly thermo-tolerant monomeric and trimeric RBD derivatives [10,11,12] that can withstand 100 °C for 90 min and 37 °C for four weeks has the potential to ease logistical burdens related to the cold-chain storage and shipping requirements of existing vaccines significantly. However, the temperature stability of ‘warm’ vaccines is of little importance if they are not sufficiently immunogenic.

In previous work [11,12], we demonstrated that serum samples from mice vaccinated with different formulations of this ‘warm’ vaccine were able to neutralise Alpha, Beta, Gamma and Delta (which were the known VOC at that time). As Omicron is fast replacing all past VOC, including Delta, which is still prevalent, we chose to compare the neutralising antibody responses of polyclonal mouse antisera generated against these thermotolerant vaccine candidates against Delta, Omicron and a reference isolate used in our previous work (VIC31-D614G) [24]. 

In line with recent findings from studies using monoclonal antibodies, mAb cocktails or patient sera, there was a significant reduction in neutralising ability of the serum collected from immunised mice against the Omicron (BA.1.1) variant compared to VIC31-D614G and Delta. In fact, the level of neutralising antibodies to Omicron was below the detection limit of the assay in a large proportion of samples, particularly those from trimeric antigen-adjuvant formulation groups. Due to low sample volumes available from the mice in this study, the high detection limit of the VNT assay used has to be acknowledged.

It appears that monomeric antigen-adjuvant formulations elicited better neutralising antibody responses compared to trimeric formulations, as demonstrated by the statistically significantly higher neutralising titres to Delta and Omicron variants. These data suggest a potential advantage in pursuing the monomeric rather than trimeric formulations for Phase I clinical trials in humans. The average 14.4- or 16.5-fold reduction in neutralisation against Omicron BA.1.1 for the monomeric and trimeric formulations, respectively, compares favourably with equivalent reductions observed with leading COVID-19 vaccines [9,19]. The present neutralisation assays were carried out with sera elicited after three immunisations as sera collected after two immunisations had been exhausted. However, we previously demonstrated that neutralisation titres elicited after two and three immunisations with the stabilised monomeric RBDs were identical [11].

The proportional reduction in neutralising antibodies to Omicron (BA.1.1), compared to VIC31-D614G and Delta rather than complete immune evasion, suggests that mouse antibodies directed to very specific epitopes in the Spike protein of the WT virus are being evaded through mutations, while antibodies to other epitopes are still able to provide significant level of virus neutralisation. This supports previous findings showing complete evasion of specific candidate therapeutic monoclonal antibodies and no evasion of others [20]. Therefore, a stronger polyclonal antibody response likely contributes to protection by neutralisation through sheer numbers despite significant evasion by the Omicron variant.

In addition to efforts to induce higher antibody levels in individuals (for example through booster vaccination with homologous antigens), another approach is regular vaccine matching to current circulating variants and/or customisation of vaccine formulations to include wild-type sequences and any relevant mutations in antigenic sites. The mRBD1-3.2 + mRBD1-3.2-beta antigen used in the formulation of Vaccine 3 in this study [10,11] was prepared using wild-type and Beta variant sequences at the time when the latter was a major concern. Although not statistically significant, there was a slightly stronger neutralisation of Omicron BA.1.1 by serum samples collected from mice immunised with this antigen, compared to only the WT antigen. 

In fact, only in this group did all mice elicit a neutralising response above the detection limit of our assay against Omicron, whereas at least two mice from all other groups had responses below the assay detection limit. However, the high detection limit in our assay has to be noted and was due to the low sample volumes available from mouse experimental serum. Nevertheless, the data suggests there might be value in adapting vaccine formulations to include key mutations from emerging variants in the spike protein of vaccine antigens. Further studies are required to investigate this and consider mutations that are common to BA.1 and BA.2, as well as those unique to each sub-lineage, given that they are antigenically distinct [25].

To understand our experimental findings, it would be beneficial to take a deeper look at the mutations in Omicron and compare monomers with trimers, as discussed below. With 15 mutations, the Omicron receptor-binding domain (RBD) presents a highly altered surface compared to the Wuhan-Hu-1 reference isolate, thus, explaining the poor neutralisation response from the vaccines based on the original strain. Even the Delta variant, with only two mutations in the RBD (L452R and T478K) has significantly reduced antibody neutralisation effects, suggestive of how specific vaccine-derived antibodies can be. The mutation positions of some variants are shown in Figure 4 as snapshots from our molecular dynamics simulations.

The trimeric vaccine construct is centred with a motif, namely a disulphide linked coiled-coil domain derived from the human cartilage matrix protein (residues 298–340) (hCMP) to the N-terminus of the RBD via a L14 linker [12]. The hCMP trimerisation domain is a relatively short, disulphide-linked stretch that we previously used in the context of a safe and efficacious HIV-1 vaccine formulation in rhesus macaques [26]. We therefore also expect it to be safe in humans, although this remains to be confirmed. 

Of note, a Spike-derived vaccine formulation developed by Clover Biopharmaceuticals that contains a much larger trimerisation domain derived from the C-propeptide of human type I(α) collagen has successfully cleared Phase 1 trials and is currently being tested in a Phase 2/3 trial [27,28]. However, the monomeric formulation of the vaccine was observed to elicit a slightly superior immune response, potentially because it presents more antigenic epitopes, and this could be explained by the following *in silico* insights.

AlphaFold predictions of the trimeric construct rank several possible structures (as shown in Figure 5) with various arrangements of the RBD against the trimerised hCMP domain. One highly altered region of the Omicron BA.1.1 RBD occurs along the side face including changes G339D, S371L, S373P, S375F and N440K, which occurs at approximately a 90 degree orientation to the receptor binding face. This coincides with some of the predicted trimer stem/RBD interfaces of AlphaFold models. If this proves to be a stable arrangement, the trimeric configuration may reduce this particular RBD epitope exposure and thus elicit a limited antibody repertoire to the full RBD. 

The high degree of variation in this region of the Omicron BA.1.1 RBD may contribute a significant effect to immune evasion. It remains unclear how representative the AlphaFold prediction are or how flexible these trimeric constructs may be (and thus how much of the epitopes are presented to the immune system) within a cellular environment, once administered. However, the predictions in Figure 5E, taken together with the transmission electron micrograph presented elsewhere [12](c.f. Figure 1h in that reference), lead to a likely scenario of key epitopes being buried and inaccessible for antibody development. Other possible structures, for example Figure 5A,B, also reveal similar stearic hindrance; therefore, the lack of a definitive prediction by AlphaFold does not affect our conclusions.

## 5. Conclusions

There are two opposing effects, one being that an oligomeric presentation is likely to induce higher titres of binding and neutralising antibodies, the other being that steric hindrance of important epitopes is required for effective B-cell responses as well as the elicitation of irrelevant antibodies against the oligomerisation domain [29]. It appears that the latter effects could contribute to this host’s (mice) response to the trimeric antigen formulations; however, more studies are required with a wider range of hosts and variants before firm conclusions can be drawn. 

At this dosage, monomeric formulations appear relatively more effective at generating neutralising antibody responses against VOC and could be preferred for phased clinical trials of this vaccine in humans. The average 14.4-fold reduction in neutralisation against BA.1.1 observed here compares favourably with other COVID-19 vaccines; vaccine matching should help improve the titres against targeted variants even further. The thermostability of this vaccine and its ability to withstand transient heat shocks is particularly promising to address the vaccine inequity that affects most low- and lower middle-income countries.

## Figures and Tables

**Table 1:** Summary of raw data on neutralising antibody titres to VIC31-D614G, Delta and Omicron BA.1.1 SARS-CoV-2 variants for individual mice and assignment to the antigen-adjuvant vaccine formulation groups. The mean titres for the positive control serum used in the neutralisation assay are also provided. For a schematic diagram of the different vaccines 1–6, see Figure 2.

Vaccine	Antigen—Presentation—Adjuvant	Mice	SARS-CoV-2 Variants *		
			VIC31-D614G	Delta	Omicron
1	mRBD1-3.2—Monomer—Addavax^TM^	1.1	1810	718	180
		1.2	180	143	≤57
		1.3	3620	718	113
		1.4	5747	1140	≤57
		1.5	5747	2281	113
2	mRBD1-3.2—Monomer—SWE	2.1	1140	905	285
		2.2	1140	143	≤57
		2.3	1810	905	180
		2.4	1810	718	≤57
		2.5	1140	1437	180
3	mRBD1-3.2 + mRBD1-3.2-beta—Monomer—SWE	3.1	7241	2874	1140
		3.2	2281	1140	71
		3.3	1437	718	90
		3.4	1437	570	226
		3.5	1140	359	226
4	hCMP-mRBD—Trimer—SWE	4.1	570	285	≤113
		4.2	1810	905	≤113
		4.3	905	404	≤113
		4.4	359	≤113	≤113
		4.5	1437	1140	≤57
5	hCMP-mRBD—CHO—Trimer—SWE	5.1	718	359	≤57
		5.2	1140	226	≤57
		5.3	1140	180	90
		5.4	1810	570	113
		5.5	1810	285	≤57
6	mRBD-GlyIZ—Trimer—SWE	6.1	718	570	90
		6.2	570	359	90
		6.3	1140	453	71
		6.4	2874	285	≤57
		6.5	1810	1437	≤57
Control Serum	Positive Control		160	113	20

Note: * All live virus neutralisation studies involved sera collected on Day 57 from mice vaccinated with dose 1 (day 0), dose 2 (day 21) and dose 3 (day 42) of the respective adjuvanted antigen. SN_50_ titres are expressed as reciprocal of the neutralising titre as calculated using the Spearman–Kärber formula. Titres expressed as ≤57 and ≤113 represent analyses for which the neutralising titres were below the assay limit of detection (lowest dilution tested) of 1:80 and 1:160, respectively. For statistical and fold-change calculations, all ≤57 and ≤113 results were assigned a value of 57. See Supplementary Text B for further explanation on titre calculations.

**Table 2:** Log_2_-transformed mean neutralising antibody titres against VIC31-D614G, Delta and Omicron BA.1.1 SARS-CoV-2 variants for mice immunised with different vaccine formulations.

Variant	Estimate	Vaccine 1	Vaccine 2	Vaccine 3	Vaccine 4	Vaccine 5	Vaccine 6
VIC31-D614G	Mean	11.02	10.42	11.02	9.76	10.29	10.22
	SD ^†^	2.09	0.37	1.07	0.95	0.56	0.95
Delta	Mean	9.49	9.36	9.75	8.52	8.22	9.02
	SD	1.47	1.28	1.14	1.71	0.64	0.90
Omicron	Mean	6.56	6.96	7.69	5.83	6.16	6.16
	SD	0.72	1.06	1.57	0.00	0.47	0.33

^†^ SD = standard deviation from the mean.

**Table 3:** One-way ANOVA analysis according to different variables.

Variable	Variant of Concern	F-Value	Pr (>F) ^a^	Tukey’s HSD Post Hoc Comparison (‘Adjusted’ *p*-Value)
Antigen	VIC31-D614G	1.012	>0.100	No significant difference
	Delta	1.463	>0.100	No significant difference
	Omicron	3.771	<0.050	Antigen 2 vs. Antigen 4 & 5 *p* < 0.1
Adjuvant	VIC31-D614G	1.534	>0.100	No significant difference
	Delta	0.688	>0.100	No significant difference
	Omicron	0	>0.100	No significant difference
Presentation	VIC31-D614G	3.412	<0.010	Monomer vs. Trimer *p* < 0.1
	Delta	4.687	<0.050	Monomer vs. Trimer *p* < 0.05
	Omicron	5.294	<0.010	Monomer vs. Trimer *p* < 0.01
Vaccine	VIC31-D614G	0.939	>0.100	No significant difference
	Delta	1.131	>0.100	No significant difference
	Omicron	3.07	<0.050	Vaccine 3 vs. Vaccine 4 *p* < 0.05
Variant	Vaccine 1	10.96	<0.010	VIC31-D614G vs. Omicron *p* < 0.01 Delta vs. Omicron *p* < 0.05
	Vaccine 2	16.21	<0.001	VIC31-D614G vs. Omicron *p* < 0.01 Delta vs. Omicron *p* < 0.01
	Vaccine 3	8.63	<0.010	VIC31-D614G vs. Omicron *p* < 0.01 Delta vs. Omicron *p* < 0.1
	Vaccine 4	13.14	<0.010	VIC31-D614G vs. Omicron *p* < 0.001 Delta vs. Omicron *p* < 0.01
	Vaccine 5	68.05	<0.0001	VIC31-D614G vs. Omicron *p* < 0.0001 VIC31-D614G vs. Delta *p* < 0.001 Delta vs. Omicron *p* < 0.001
	Vaccine 6	35.66	<0.0001	VIC31-D614G vs. Omicron *p* < 0.0001 VIC31-D614G vs. Delta *p* < 0.1 Delta vs. Omicron *p* < 0.001

a—Estimated *p*-value.

## Data Availability

The original contributions presented in the study are included in the article/Supplementary Material. Further inquiries can be directed to the corresponding author.

## Supplementary Materials

The following supporting information can be downloaded at: https://www.mdpi.com/article/10.3390/v14040800/s1, Supplementary Text A: Amino Acid Sequences of Vaccine Formulations; Supplementary Text B: Complications in the Analysis of Microneutralisation Assay Data [30].
