# Supplementary material for: Highly Thermotolerant SARS-CoV-2 Vaccine Elicits Neutralising Antibodies against Delta and Omicron in Mice
Source: Viruses. 2022 Apr 13;14(4):800. doi: 10.3390/v14040800 (PMC9031315; doi:10.3390/v14040800)
Supplement: Supplementary file 1 [file viruses-14-00800-s001.zip › Supplementary Text A.pdf]

Article

Highly Thermotolerant SARS-CoV-2 Vaccine Elicits Neutralising Antibodies Against Delta and Omicron in Mice

Petrus Jansen van Vuren[1]†, Alexander J. McAuley[1]†, Michael J. Kuiper[2]†, Nagendrakumar B. Singanallur [1], Matthew P. Bruce[1], Shane Riddell[1], Sarah Goldie[1], Shruthi Mangalaganesh[1,3], Simran Chahal[1], Trevor W. Drew[1], Kim R. Blasdell[1], Mary Tachedjian[1], Leon Caly[4], Julian D. Druce[4], Shahbaz Ahmed[5], Mohammad Suhail Khan[5], Sameer Kumar Malladi[5], Randhir Singh[6], Suman Pandey[6], Raghavan Varadarajan[5] and Seshadri S. Vasan[1,7,*]

Supplementary Text A

Amino Acid Sequences of Vaccine Formulations

Vaccine 1/2: mRBD1-3.2 (Monomer)

EISNITNLCPFGEVFNATRFPSVYAWNRKRISNCVADWSVLYNSASFSTFKCYGVSPTK
LNDLCFTNVYADSFVIRGDEVRQIAPGQTGKIADYNYKLPDDFTGCVIAWNSNNLDS
KVGGNYNYLYRLFRKSNLKPFERDISTEIYQAGSTPCNGVEGFNCYFPLQSYGFQPTN
GVGYQPYRVVVLSFELLHAPATVCGLKKSTNGSLEVLFQ

Vaccine 3: mRBD1-3.2-beta (Monomer)

EISNITNLCPFGEVFNATRFPSVYAWNRKRISNCVADWSVLYNSASFSTFKCYGVSPTK
LNDLCFTNVYADSFVIRGDEVRQIAPGQTG**N**IADYNYKLPDDFTGCVIAWNSNNLDS
KVGGNYNYLYRLFRKSNLKPFERDISTEIYQAGSTPCNGV**K**GFNCYFPLQSYGFQPT**Y**
GVGYQPYRVVVLSFELLHAPATVCGLKKSTNGSLEVLFQ
(Red residues represent mutations relative to Wuhan-Hu-1)

Vaccine 4/5: hCMP-mRBD (Trimer)

EISEEDPCACESLVKFQAKVEGLLQALTRKLEAVSKRLAILENTVVASSEGTMMRGEL
KNITNLCPFGEVFNATRFASVYAWNRKRISNCVADYSVLYNSASFSTFKCYGVSPTKL
NDLCFTNVYADSFVIRGDEVRQIAPGQTGKIADYNYKLPDDFTGCVIAWNSNNLDSK
VGGNYNYLYRLFRKSNLKPFERDISTEIYQAGSTPCNGVEGFNCYFPLQSYGFQPTNG
VGYQPYRVVVLSFELLHAPATVCGPKKSTNGSLEVLFQ
(Orange residues are hCMP; blue residues are the RBD)

Vaccine 6: mRBD-GlyIZ (Trimer)

EISITNLCPFGEVFNATRFASVYAWNRKRISNCVADYSVLYNSASFSTFKCYGVSPTKL
NDLCFTNVYADSFVIRGDEVRQIAPGQTGKIADYNYKLPDDFTGCVIAWNSNNLDSK
VGGNYNYLYRLFRKSNLKPFERDISTEIYQAGSTPCNGVEGFNCYFPLQSYGFQPTNG
VGYQPYRVVVLSFELLHAPATVCGPKKSTNAGSGSGSGSAGSNGTGRMKQIEDKIENI
TSKIYNITNEIARIKKLIGNRTAS
(Orange residues are GlyIZ; blue residues are the RBD)
