# Supplementary material for: Highly Thermotolerant SARS-CoV-2 Vaccine Elicits Neutralising Antibodies against Delta and Omicron in Mice
Source: Viruses. 2022 Apr 13;14(4):800. doi: 10.3390/v14040800 (PMC9031315; doi:10.3390/v14040800)
Supplement: Supplementary file 1 [file viruses-14-00800-s001.zip › Supplementary Text B.pdf]

Article

Highly Thermotolerant SARS-CoV-2 Vaccine Elicits Neutralising Antibodies Against Delta and Omicron in Mice

Petrus Jansen van Vuren[1]†, Alexander J. McAuley[1]†, Michael J. Kuiper[2]†, Nagendrakumar B. Singanallur [1], Matthew P. Bruce[1], Shane Riddell[1], Sarah Goldie[1], Shruthi Mangalaganesh[1,3], Simran Chahal[1], Trevor W. Drew[1], Kim R. Blasdell[1], Mary Tachedjian[1], Leon Caly[4], Julian D. Druce[4], Shahbaz Ahmed[5], Mohammad Suhail Khan[5], Sameer Kumar Malladi[5], Randhir Singh[6], Suman Pandey[6], Raghavan Varadarajan[5] and Seshadri S. Vasan[1,7,*]

Supplementary Text B

Complications in the Analysis of Microneutralisation Assay Data

1. Handling of Data Below or Above the Limit of Detection (LOD)

Given that sample volumes are frequently low, especially when collected from small animal models such as mice, dilution series often need to be started at suboptimal sample dilutions. This poses problems for the analysis and interpretation of data, e.g., lack of neutralisation at a starting dilution of 1:80 does not mean that the sample contains no neutralising antibodies. Convention would have an arbitrary value assigned to the sample in this situation, however the particular arbitrary value used can substantially affect the calculation of fold-differences and statistical analysis. Not having a standardised approach across different research groups will make it difficult for the WHO and other public health bodies to make meaningful comparisons and policy decisions [30].

The conservative approach would be to assign a value one dilution factor lower than the lower LOD (or one dilution factor higher than the upper LOD) for any sample for which a quantifiable titre cannot be obtained. Although this approach will frequently overstate neutralisation of low-responding samples, this will result in a sample below the lower LOD (BLOD) having the maximum possible neutralisation titre, and a sample above the upper LOD having the minimum possible neutralisation titre. Accordingly, calculated fold change differences between groups will be the minimum, and statistical comparison will be most conservative.

Example 1: Low sample volume availability means that four samples have to be used at a starting dilution of 1:40. Each sample is run in triplicate.

Results

Sample 1: 80, 160, 160
Sample 2: BLOD, BLOD, BLOD
Sample 3: BLOD, 40, 40
Sample 4: BLOD, 40, BLOD

Analysis

Sample 1: An NT_{50} value can be calculated using the Spearman-Karber approach as 180
Sample 2: All samples are below the LOD (no neutralisation at 1:40). Arbitrary value set at one dilution below, i.e., replicate titres 20, 20, 20. NT_{50} value calculated as 28
Sample 3: One sample below LOD, two have neutralisation at 1:40. Arbitrary value set at one dilution below, i.e., replicate titres 20, 40, 40. NT_{50} value calculated as 45
Sample 4: Two samples below LOD, one has neutralisation at 1:40. Arbitrary value set at one dilution below, i.e., replicate titres 20, 40, 20. NT_{50} value calculated as 36

2. Handling of Data Below the LOD with Different Starting Dilutions

Where possible, samples should be run using the same starting dilution, however occasionally it is necessary to balance assay resolution with sample availability. If comparisons are to be made between such samples, every effort should be made to minimise the difference between the starting dilutions (ideally no more than one dilution factor different). For titres that can be calculated from the dilution series, there will be no issues with the difference in starting dilution, however if samples have titres below the LOD there will be complications. In this situation, the arbitrary value assigned to all samples below the LOD should be that of the lower starting dilution. The exception being if one or more replicates have neutralisation at the higher starting dilution. In this situation, an arbitrary value one dilution lower than that sample's starting dilution will be used.

Example 2: Low sample volume for some of the samples means that two samples can be used at a starting dilution of 1:10, while two samples have to be started at 1:20. Each sample is run in triplicate.

Results

Sample 1 (starting dilution 1:10): 20, 40, 20
Sample 2 (starting dilution 1:10): BLOD, BLOD, BLOD
Sample 3 (starting dilution 1:20): BLOD, BLOD, BLOD
Sample 4 (starting dilution 1:20): 20, BLOD, 20

Analysis

Sample 1: An NT_{50} value can be calculated using the Spearman-Karber approach as 36
Sample 2: All samples are below the LOD (no neutralisation at 1:10). Arbitrary value set at one dilution below, i.e., replicate titres 5, 5, 5. NT_{50} value calculated as 7
Sample 3: All samples are below the LOD (no neutralisation at 1:20). Arbitrary value set at one dilution below lowest starting dilution used in assay (1:10), i.e., replicate titres 5, 5, 5. NT_{50} value calculated as 7
Sample 4: One samples below LOD, two have neutralisation at 1:20. Arbitrary value set at one dilution below sample starting dilution, i.e., replicate titres 20, 10, 20. NT_{50} value calculated as 22
